# Automated Extraction of Dose/Volume Statistics for Radiotherapy-Treatment-Plan Evaluation in Clinical-Trial Quality Assurance

**DOI:** 10.3389/fonc.2016.00047

**Published:** 2016-03-03

**Authors:** Yutao U. T. Gong, Jialu Yu, Dalong Pang, Heming Zhen, James Galvin, Ying Xiao

**Affiliations:** ^1^IROC, Philadelphia, PA, USA; ^2^Department of Radiation Medicine, Georgetown University Hospital, Washington, DC, USA; ^3^Rush University Medical Center, Chicago, IL, USA; ^4^Department of Radiation Oncology, University of Pennsylvania, Philadelphia, PA, USA

**Keywords:** radiation therapy plan evaluation, clinical trial, quality assurance, automated data processing, Matlab

## Abstract

Radiotherapy clinical-trial quality assurance is a crucial yet challenging process. This note presents a tool that automatically extracts dose/volume statistics for determining dosimetry compliance review with improved efficiency and accuracy. A major objective of this study is to develop an automated solution for clinical-trial radiotherapy dosimetry review.

## Introduction

Quality assurance (QA) of radiation therapy (RT) procedures that include treatment-plan review is an important mechanism to ensure adequate dose coverage for tumor(s), while identifying risks of normal tissue complications (1–3). The Imaging and Radiation Oncology Core (IROC) provides RT QA services to the National Clinical Trial Network (NCTN). IROC services include site qualification, trial-design support, credentialing, data management, and case review (4–6). Case review includes a data-integrity review, a review of compliance with target volume and organs at risk contours by study chairs, and a review of dosimetry compliance (5). The dosimetry review (4, 7–11) checks whether the data points on planned structure dose–volume histograms (DVH) meet the protocol criteria. Non-compliance of target volume–dose criteria may result in treatment failure, and violation of organs at risk criteria may create complications and toxicities. It is a consensus that timely clinical-trial data QA is crucial for the successful RT clinical-trial management (4, 7, 8, 10, 12–15). A study of 174 medulloblastoma patients in 1999 found that the number of major target deviations in RT was strongly correlated with the risk of tumor relapse (16). Another study of 416 pancreatic cancer patients found that failure to adhere to protocol-specified radiation-therapy guidelines was associated with decreased survival (17). One published meta-analysis about radiotherapy-protocol deviations and clinical outcomes (18) included two lung cancer trials (19, 20), three trials for medulloblastoma or supratentorial primitive neuroectodermal tumors (16, 21, 22), and one trial each for Ewing sarcoma (23), pancreatic cancer (17), and head and neck cancer (7). The study found the frequency of RT QA deviations ranged from 8 to 71% (median 32%), and RT deviations were associated with a statistically significant decrease in overall survival and also secondary outcomes (18).

Automatic data processing is needed to improve the efficiency of RT QA, which at present is a crucial yet labor-intensive and challenging task (4). Uniformity in the data format used for information collection is a prerequisite of automatic data processing. Recently, infrastructures and guidelines were developed to improve the data uniformity within NCTN (24, 25). A submission-ready case should have all the structures required by the protocol, and the structure names should exactly follow the standard names as stated in each protocol (24). The dosimetry compliance review usually is a task of checking more than 10-dose/volume points for agreement with stated protocol limits (Table 1). Considering that typically 100 or more patients are to be recruited per trial and there are more than 50 RT open trials on the NRG Oncology website (26), a tool that automatically extracts dose–volume points for dosimetry compliance review will save time, improve accuracy, and catch underlying issues with understanding of the protocol text. In this report, we introduce an automated solution that meets the need for rapid dosimetry review and data extraction.

**Table 1:** **Dosimetry-review workload of selected NRG trials (26)**.

Trial	No. of structures requiring review	No. of data points to be reviewed	Target accrual for trial
HN001	17	18	758
HN002	9	14	296
GU001	3	5	185
LU001	6	14	168
BN001	13	15	576
GI001	10	16	182

## Methods

### Workflow of the Solution

**Figure 1:**
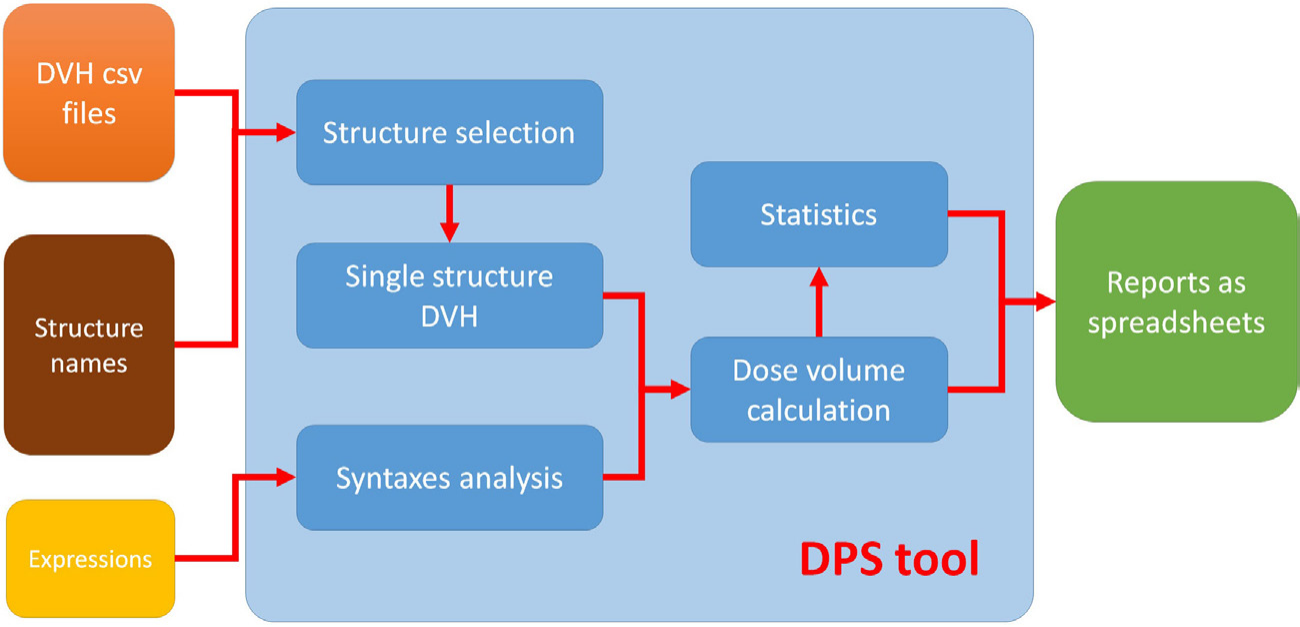
**Typical work flow of DPS tool**.

The proposed automated solution to QA for clinical-trial data management consists of three separate parts: (i) a standard syntax system that defines the plan–evaluation data points, (ii) an automated program with graphic user interface (GUI) written in MATLAB, and (iii) a result output MATLAB program that sorts and lists the results as a spreadsheet. The combination of the MATLAB programs is called dose/volume point statistics (DPS) (27). The workflow of this solution is illustrated in Figure 1. DPS takes user input (structure names and expressions of dose–volume points) and DVH files from DVH reviewer of MIM (MIM Software Inc., Cleveland, OH, USA) and automatically completes computation and statistics, and then generates reports. The DVH files referred to in this report use the comma-separated value format and contain more information than the simple DVH values (see Format of DVH File for more detailed information).

**Figure 2:**
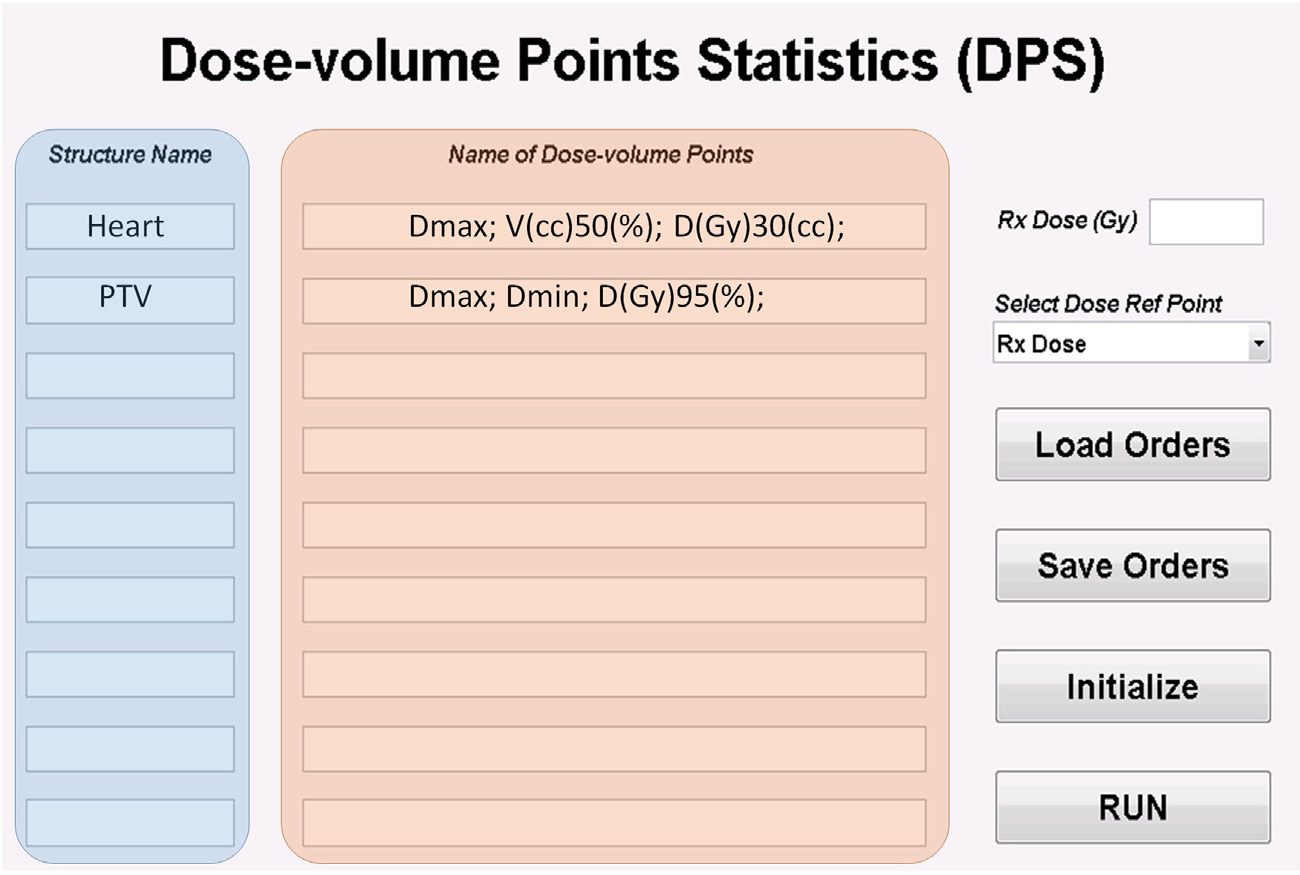
**Panel of dose/volume points statistics (DPS)**.

The GUI for DPS is shown in Figure 2. The data extraction requests are specified by entering the structure names and names of dose/volume points in DPS. The request can be exported and imported as a plain text file. The run button in GUI loads the DVH files. Details for preparing the structure names, naming of both dose/volume points and DVH files can be found in Sections “Structure Names,” “Standard Syntax System,” and “Format of DVH File.” For calculating the normalized dose (e.g., the ratio of max dose to the prescription dose), a global dose reference point is set. DPS allows two choices of the dose reference point: the prescription dose and the maximum dose. The prescription dose can be entered in DPS as a global parameter for all cases; however, the maximum dose must be from the individual DVH files. DPS is designed to accommodate protocols with variable prescription doses. A specific prescription dose value saved in the DVH file overwrites the globally defined prescription dose.

**Figure 3:**
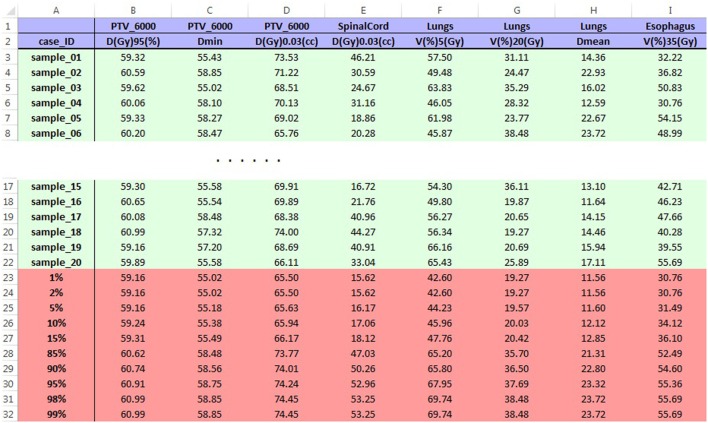
**Sample report spreadsheet for LU001 opened in Excel**. The blue region on the top shows dosimetry-review requests entered in GUI, and the green region contains values of dose–volume points calculated by DPS. The red region is the statistics of values in the green region. Data shown in the table are made examples and are not from submitted cases.

In operation, DPS extracts DVH data of the structure in the first row of GUI from the first DVH files, and it calculates values of the dose/volume points in the same row of GUI. Then, DPS moves to the next row of GUI until all dosimetry-review requests are processed for the case and moves on to the next case. When all cases are processed, DPS tabulates the dose/volume points for all cases in one spreadsheet report and calculates 1, 2, 5, 10, 15, 85, 90, 95, 98, and 99% quantiles of each dose/volume point (Figure 3). This report can be used to evaluate the distribution of plan qualities for a particular protocol. Based on this information, it is possible to make the decision to amend a protocol to improve the performance of institutions’ enrollments.

### Structure Names

Although DPS accepts and processes any structure names defined by users, in order to avoid errors and confusion it is highly recommended to name the structures according to the consensus of the NCTN structure-naming convention defined by Yu et al. (24). While processing a large number of cases, maximum performance of DPS is achievable by sorting case data by different arms of a protocol and running DPS separately for each arm.

### Standard Syntax System

Dosimetry-review data points specified in protocols can be entered in DPS using a standard syntax system (Table 2). This syntax system intends to avoid possible confusion between absolute values and relative values, as well as the inconvenience of using subscripts. DPS checks syntax on all the input expressions and reminds users of any non-acceptable expressions.

**Table 2:** **Examples of expressions of dose/volume points and their definitions**.

Expressions	Definition
D(Gy)40(cc)	The dose in Gy that covers 40 cc of the structure
D(Gy)40(%)	The dose in Gy that covers 40% of the structure
D(%)40(cc)	The dose in percentage that covers 40 cc in the structure
D(%)40(%)	The dose in percentage that covers 40% of the structure
V(cc)40(Gy)	The volume in cc that is covered by a dose not <40 Gy
V(cc)40(%)	The volume in cc that is covered by a dose not <40% of the reference dose
V(%)40(Gy)	The volume in percentage that is covered by a dose not <40 Gy
V(%)40(%)	The volume in percentage that is covered by a dose not <40% of reference dose
Dmax	Maximum dose defined by RTOG, the same as D(Gy)0.03(cc)
Dmin	Minimum dose defined by RTOG, the lowest dose for a point in the volume that is at least 0.03 cc in size (found on the DVH curve at a volume that is the total volume of the structure – 0.03 cc)
Dmean	Mean dose in the structure
Dmean/Rx	Mean dose divided by the prescription dose
Dmean/Rx × 100	Mean dose divided by the prescription dose and multiplied by 100

Generally, in the proposed syntax system, “D” stands for dose with the units (Gy or %) defined in the following pair of parentheses, whereas “V” stands for volume with the units (cc or %). Table 2 describes the syntaxes and their definitions. After setting the dose reference point, which is set to be 100% of the relative dose, the user is able to calculate percentage dose to meet special needs. Popular dose reference points include prescription dose and maximum dose. In addition, simple calculation is supported by the syntax system. For example, Dmean/Rx is the ratio of mean dose to prescription dose.

### Format of DVH File

**Figure 4:**
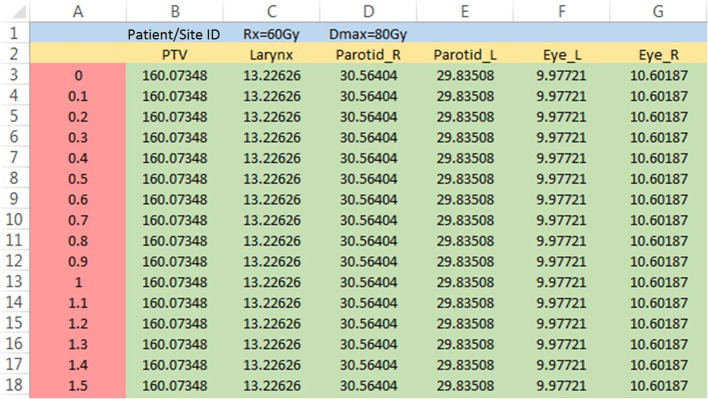
**An example DVH csv file opened in Excel**. The file is partially displayed.

For NRG, all the RT data for submission-ready cases are saved in commercial software for review (MIMvista, Cleveland, OH, USA). The structure-dosimetry data for each case can be exported as a DVH file that is written in a comma-separated value format. These files are sometimes identified as DVH csv files. As shown in Figure 4, the first line (blue region) contains the patient/site ID, and the second line (yellow region) has structure names. Numeric data are stored at line 3 and below, in which the first column is the dose data (red region), and the rest (green region) contains the absolute volume data of different structures.

As mentioned in Section “Workflow of the Solution,” DPS accommodates protocols with variable prescription dose. Case-specific prescription dose can be saved in cell C1 of the DVH file (“Rx = 60 Gy,” or in short, “60”). When dose reference point for dosimetry review is set to be the global maximum dose, the maximum dose for each case needs to be saved in cell D1 of the DVH csv file (“Dmax = 80 Gy,” or in short, “80”), since different plans usually do not share the same global maximum dose.

### Performance of DPS

To test the performance of this tool, data extraction for dosimetry review of 20 DVH csv files was performed using NRG RT trial LU001 protocol (28). The time needed to process the task with DPS is recorded. As a comparison of the efficiency, the same task is repeated in a manual way by reading dose/volume points from MIMvista and typing values in a spreadsheet.

## Results

To test the performance of this tool, data extraction for dosimetry review of 20 DVH csv files was performed using NRG RT trial LU001 protocol (28). Including the time for user operation such as selecting DVH files, it took DPS 3 min to generate the final report in Excel with the computer setting as Windows 7 Professional, Intel Xeon CPU 3.20 GHz, and 12.0 GB RAM. The report includes a spreadsheet for each structure as well as an overview spreadsheet for all the structures (Figure 3). The statistical results were included at the end of each spreadsheet. It took more than 1 h to manually extract and tabulate the same dose/volume points, not including the time of loading the case in DVH viewer of MIMvista.

## Discussion

The DPS tool shows great capability of efficiency on dosimetry reviews by automating data extraction. The data extraction request specifications could be exported and imported, thus one can perform the same analysis for future DVH csv files or as needed, which improves task consistency and documentation completeness.

The report from the DPS tool can be used to evaluate the distribution of plan qualities for a particular protocol. Based on this information, it is possible to make the decision to amend a protocol to improve the performance of institutions registering patients. In addition, DPS allows for comparing different ways to evaluate the dosimetry. For example, structure maximum dose can be in the formats of D(Gy)1(%)/Rx, D(Gy)1(%), and D(Gy)0.03(cc). One can add all the three formats of maximum doses to DPS, and the results in the three formats will be tabulated together, which is a helpful feature for protocol development. Furthermore, with minor revision, DPS may perform biomathematical NTCP and TCP model calculations, which will help to understand the biological outcomes of the cases.

## Conclusion

With the infrastructures and structure name guidelines adopted for clinical-trial data management (24, 25), automated tools, such as DPS, can greatly improve the efficiency and organization of clinical-trial dosimetry review.

## Author Contributions

All authors contributed to manuscript writing/revisions. YG, JY, HZ, and YX contributed to development of the tool/method. YG, JY, and YX contributed to tool testing. DP and JG made important revisions to the manuscript to achieve publishable quality.

## Conflict of Interest Statement

The authors declare that the research was conducted in the absence of any commercial or financial relationships that could be construed as a potential conflict of interest.
